# The New Metal Gallium

**Published:** 1876-08

**Authors:** 


*The New Metal Gallium.*—The properties of this new metal are described as follows in the *Med. and Surg. Reporter*:

The recently discovered metal gallium melts at 85.1° Fah., so that it liquifies when held in the hand. When solid, the metal is hard and resistant, even to a few degrees below the melting point. It can be cut, and possesses a slight malleability. When fused, it adheres easily to glass, on which it forms a beautiful mirror, whiter than that produced by mercury, It oxidizes but very superficially when heated to redness in the air, and does not become volatile. The density at 50° Fah. is 4.7 that of water at 39.2° Fah. being one.

Excepting mercury, which only becomes solid at 47.9° Fah., there is no other element which liquifies at so low a temperature as gallium.

